# Bone marrow transplant model to study articular cartilage repair

**DOI:** 10.1186/1476-9255-12-S1-P9

**Published:** 2015-04-16

**Authors:** Ana Sergijenko, Anke J Roelofs, Andrea Augello, Cosimo De Bari

**Affiliations:** 1 Regenerative Medicine Group, Musculoskeletal Research Programme, University of Aberdeen, Scotland, AB25 2ZD, UK

## 

We have previously established a mouse model of spontaneous cartilage repair following a full thickness injury and are studying the contribution of mesenchymal stem cells (MSCs) to this process. To determine the contribution of MSCs from bone marrow, we developed a chimeric model by transplanting eGFP+ bone marrow cells into lethally myeloablated wild-type mice. Stromal cell chimerism was confirmed in bone marrow by flow cytometry based on the CD45-/Sca1+/Pdgfr-α+/GFP+ selection and CFU-F assay. GFP+ cells were first detected in synovium at 7 days post-transplant (5%), which increased to 40% of nucleated cells at 8 weeks. GFP+ cells in the synovium were positive for stromal cell markers CD44 and cadherin11, but not endothelial cell markers. To further validate this model, we assessed the response of donor cells to cartilage injury in the synovium post-transplant. Chimeric mice underwent joint surface injury and immediately received a nucleoside analogue chlorodeoxyuridine (CldU) in water for 7 days, after which mice were analysed. There was an increase of donor-derived GFP+ cells in synovium of injured mice compared to uninjured, however, the number of proliferating cells (GFP+/ CldU+) did not increase significantly, suggesting that bone marrow derived cells do not proliferate in situ, but rather infiltrate from bone marrow. In conclusion, we established a chimeric mouse model that will allow us to track contribution of bone marrow cells to cartilage repair.

